# Critical field enhancement of asymptotic optical bound states in the continuum

**DOI:** 10.1038/srep18301

**Published:** 2015-12-17

**Authors:** Jae Woong Yoon, Seok Ho Song, Robert Magnusson

**Affiliations:** 1 Department of Electrical Engineering, University of Texas - Arlington, Box 19016, Arlington, TX 76019, USA; 2 Department of Physics, Hanyang University, Seoul, 133-791, KOREA

## Abstract

We study spectral singularities and critical field enhancement factors associated with embedded photonic bound states in subwavelength periodic Si films. Ultrahigh-Q resonances supporting field enhancement factor exceeding 10^8^ are obtained in the spectral vicinity of exact embedded eigenvalues in spite of deep surface modulation and vertical asymmetry of the given structure. Treating relations between the partial resonance Q and field enhancement factors with an analytical coupled-mode model, we derive a general strategy to maximize the field enhancement associated with these photonic bound states in the presence of material dissipation. The analytical expression for the field enhancement quantitatively agrees with rigorous numerical calculations. Therefore, our results provide a general knowledge for designing practical resonance elements based on optical bound states in the continuum in various applications.

High-Q optical resonances in periodic thin films are connected conceptually to embedded bound states which do not reradiate external waves. These were originally proposed in hypothetical quantum systems by von Neumann and Wigner^1^. In such systems, a completely bound state exists at an energy level above the lowest continuum level. Marinica *et al.* proposed a symmetric double-grating structure to support embedded photonic bound states by coupling between two identical resonant grating layers^2^. Hsu *et al.* experimentally showed a diverging radiation Q factor as a signature of embedded bound states in a single layer of a freestanding Si_3_N_4_ thin-film photonic crystal^3^. More recently, complete 3-dimensional optical confinement in open scattering systems was suggested using layered plasmonic nanoparticles^4^,^5^.

In real optical systems, embedded bound states are excited or probed in an asymptotic manner^3^,^6^ since at the exact condition they do not interact with the continuum states that contribute to the observation in the far field. In other words, what we actually measure in the laboratory is the resonance response of leaky modes that asymptotically approach a targeted bound state creating a virtual singularity in the spectrum. In this aspect, an important question concerns the properties of the resonance Q factor (*Q*) as the configuration approaches the exact bound state condition. A related point is the maximal achievable field enhancement. Obviously, field enhancement or excitation strength does not simply increase with diverging *Q* because the mode excitation vanishes at the exact point of *Q* = ∞ as long as no significant non-linear coupling occurs between the bound state and the external continuum^7^. Therefore, there must exist an optimum quasi-embedded bound-state resonance configuration somewhere in-between highly leaky and completely trapped mode conditions. This is a crucial problem for sensing, nonlinear applications, and cavity-QED problems. Nevertheless, this issue has not been discussed in detail to date. Importantly, leaky-mode resonance with intermediate *Q* and associated strong field enhancement is currently of high interest as they have versatile properties enabling high-performance optical filters^8^, label-free biosensors^9^, lossless mirrors^10^, dielectric metasurfaces^11^,^12^, dielectric-based optical magnetism^13^, and many others^14^,^15^.

In this paper, we study resonance Q factors and field enhancement effects originating in embedded photonic bound states in periodic Si thin-film structures. We show that a 700-nm-thick fully etched Si thin-film grating supports a symmetry-protected bound state at zero in-plane wave vector (*k*_*||*_ = 0) with ultrahigh resonance Q and electric field intensity enhancement factors exceeding 10^8^. The same structure also supports a quasi-embedded bound state at *k*_*||*_ ≠ 0 in spite of vertical asymmetry of the structure. Field enhancement factors of these photonic resonances show a non-trivial relation with the resonance Q factors in the presence of absorption. We explain this relation with an analytic theory based on the coupled-mode theory of optical Fano resonances. As a crucial factor for applying this device class to practical applications, we discuss methods to maximize resonance Q factors for a given, unavoidable level of material dissipation.

## Spectral and angular properties

**Figure 1:**
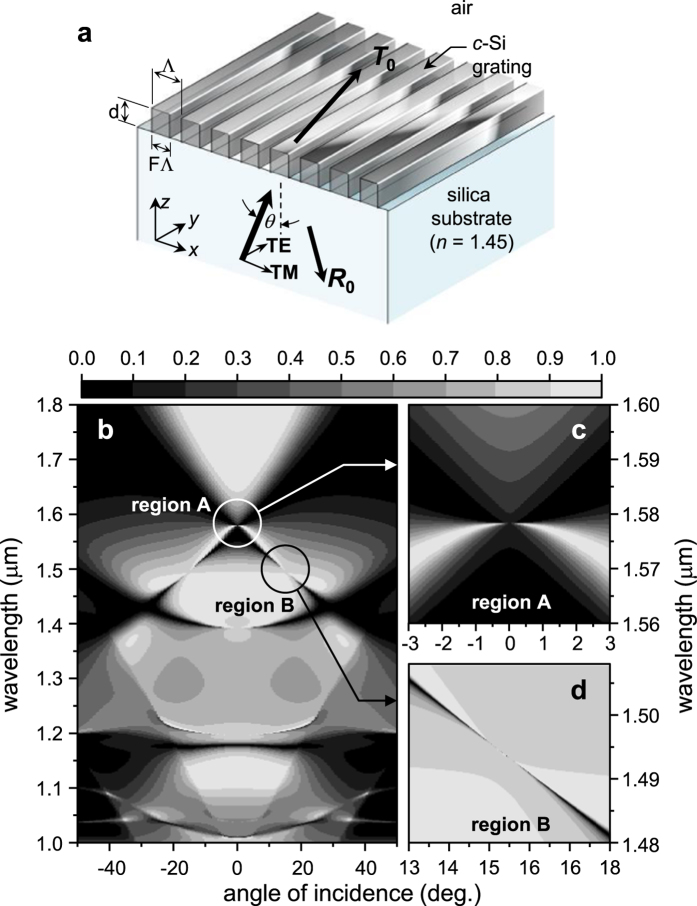
Geometry of the problem and spectral response of an exemplary structure. (**a**) Schematic of a high-Q resonance element consisting of a fully etched crystalline Si (*c*-Si) grating on SiO_2_ substrate. (**b**–**d**) Optical response of a *c*-Si thin-film grating with Λ = 660 nm, F = 0.7, and d = 700 nm under TM-polarized light incidence. (**b**) Zero-order transmittance (*T*_0_) as a function of wavelength and angle of incidence. (**c**,**d**) show magnified *T*_0_ maps for regions A and B, respectively.

We consider a subwavelength periodic thin-film structure consisting of a crystalline Si grating with period Λ, fill factor F, and thickness d between an air half space and a silica substrate (*n*_sub_ = 1.45) as shown in Fig. 1a. Polarization of the incident optical field is defined in reference to the plane of incidence: transverse electric (TE) polarization for the electric field perpendicular to the plane of incidence and transverse magnetic (TM) polarization for the magnetic field perpendicular to the plane of incidence. *θ* denotes the angle of incidence. We use the rigorous coupled-wave analysis^16^ for numerical calculation of the spectral response and associated internal field properties. We model the crystalline Si with frequency dispersive complex refractive index *n*_Si_(λ) = *n*_R_(λ) + *ik*_I_(λ) and use *n*_R_ and *k*_I_ values listed in^17^.

**Figure 2:**
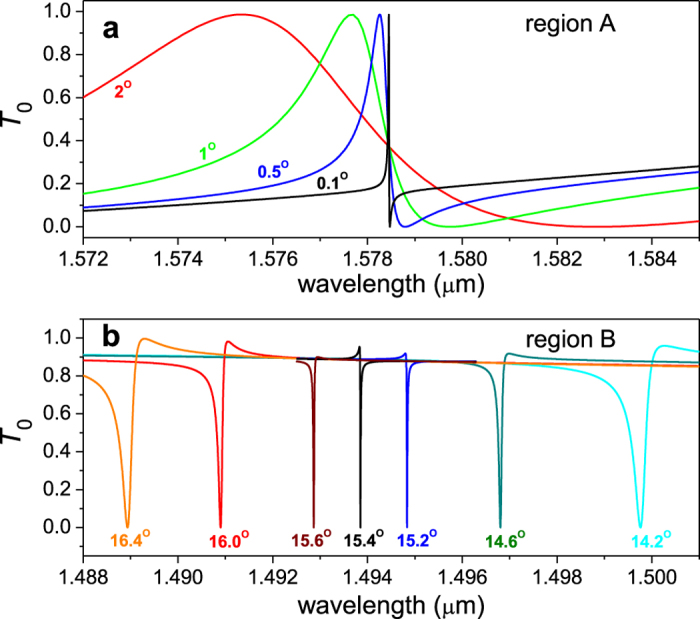
Spectral profiles of zero-order transmittance for several sampled angles of incidence in region A (**a**) and region B (**b**).

Figure 1b shows the angle-dependent zero-order transmission (*T*_0_) spectrum through a c-Si thin-film grating with Λ = 660 nm, F = 0.7, and d = 700 nm under TM-polarized plane-wave incidence. The *T*_0_ spectrum shows broad high and low transmission regions accommodating many sharp resonance features. Here, we focus on the sharp resonance features for further analysis. The two features in regions A and B show vanishingly narrow resonance bandwidth as *θ* approaches 0° and 15.4°, respectively. These regions are magnified in Fig. 1c,d for clearer confirmation of the vanishing bandwidth. These high-Q resonance excitations produce extremely sharp spectral profiles as shown in Fig. 2. The resonance bandwidth approaches 2.9 fm for the region-A resonance and 9.2 pm for the region-B resonance. The corresponding resonance Q-factors are 5.46 × 10^8^ and 1.62 × 10^5^ for resonance in region A and B, respectively.

**Figure 3:**
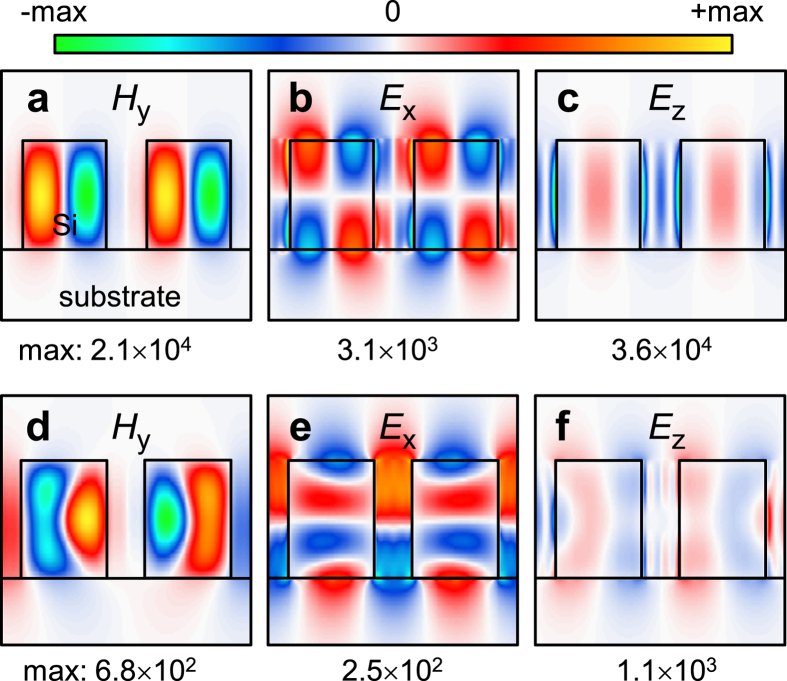
Field distributions associated with the high-Q resonance features in (**a–c**) region A and (**d–f**) region B. Corresponding wavelengths and angles of incidence are selected when the field enhancement becomes maximal. For (**a**–**c**), λ = 1578.457 nm and *θ* = 0.00125°. For (**d**–**f**), λ = 1493.849 nm and *θ* = 15.4°. Note *H* and *E* denote magnetic and electric field amplitudes, respectively, and the values are normalized by incident field amplitudes.

Internal field distributions reveal the nature of the modes inducing these high-Q resonances. The field distributions associated with the region-A resonance are shown in Fig. 3a–c when the field enhancement becomes maximal at λ = 1578.547 nm and *θ* = 0.00125°. We will explain the reason why the field enhancement maximum occurs at this particular angle of incidence later in this paper. In these field distributions, we note that the electric field intensity |**E**|^2^ inside the Si ridges and their surfaces is enhanced by a factor exceeding 10^8^. Hence this resonance excitation is highly desirable for surface-emitting Si-Raman amplifiers/lasers. In addition, it is of great interest to optimize the proposed device class in liquid environment for its application as efficient surface-enhanced Raman templates where at least 10^7^–10^8^ scale electric field enhancement factor is required for single-molecule-level detection sensitivity^18^. The field distributions show that each Si ridge excites a typical radiation patterns of a cylindrical electric dipole aligned with surface normal axis. The tangential field components (*H*_*y*_ and *E*_*x*_) are anti-symmetric with respect to the mirror-symmetry plane of the structure, i.e., *y*-*z* plane in our case. Therefore, leakage radiation to the surface normal direction is forbidden because of symmetry incompatibility with the external radiation whose tangential field components are symmetric with respect to the mirror-symmetry plane. Leakage radiation to the off-normal direction is also forbidden due to the subwavelength periodicity. This property explains the vanishingly narrow resonance bandwidth of the region-A resonance as *θ* → 0°. This type of radiation decay suppression is also closely related to destructive interference between emitted radiation fields from two counter-propagating leaky guided modes for standing-wave conditions at either one of two edges of a stopband^19^,^20^.

In contrast, the region B resonance is not explained by the mode’s symmetry incompatibility with the external radiation fields or interference properties for standing-wave conditions because the resonance bandwidth vanishes for non-zero angle *θ* → 15.4° and the incident fields are not necessarily symmetric or anti-symmetric with respect to the mirror-symmetry plane of the structure. Instead, the physical origin of the ultrahigh-Q resonance in region B is related to simultaneous suppression of the leakage radiation amplitudes to the zero-order waves in air cover and silica substrate. This effect involves interference between partial leakage radiations from different Bloch modes and complex interaction of evanescent fields at the top and bottom interfaces of the film^3^,^21^. The internal field distributions associated with the region B resonance are shown in Fig. 3d–f and we confirm the electric field intensity enhancement factor in the order of 10^5^.

## Resonance Q factors and field enhancement

Considering applications of high-Q resonances in periodic thin films to biochemical sensors, SERS templates, and nonlinear optical components, the resonance Q factor *Q* and associated field intensity enhancement factor *W* are important figures. We further investigate dependences of these parameters on the excitation condition and derive a useful relation between *Q* and *W* in the presence of material dissipation that is unavoidable due to internal absorption and diffuse scattering by film imperfections such as surface roughness, vacancies, and grain boundaries.

Following the conventional terminology, we define the total resonance Q factor *Q*_tot_ ≡ λ_0_/Δλ = *ω*_0_/2(*γ*_A_ + *γ*_R_), where λ_0_ is resonance center wavelength, Δλ is full-width at half-maximum of the resonance, *ω*_0_ is angular frequency of the resonance center, and *γ*_A_ and *γ*_R_ denote partial damping rates due to the material dissipation and leakage radiation, respectively. We also define partial resonance Q factors *Q*_A_ ≡ *ω*_0_/2*γ*_A_ as dissipation Q factor and *Q*_R_ ≡ *ω*_0_/2*γ*_R_ as radiation Q factor. The total and partial Q factors are related by 1/*Q*_tot_ = 1/*Q*_A_ + 1/*Q*_R_. For quantitative analysis of the field enhancement effect, we define two electric field intensity enhancement factors


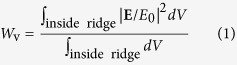


for the volume-average intensity inside the Si ridges and


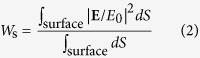


for the surface-average intensity on the surface exposed to the air, where **E** denotes electric field, *E*_0_ is incident electric field amplitude, *dV* is infinitesimal volume, and *dS* is infinitesimal surface area. The two enhancement factors *W*_V_ and *W*_S_ are useful parameters in consideration of a resonance element as a nonlinear optical device using the film’s native optical nonlinearity and a SERS template for molecular detection systems, respectively.

**Figure 4:**
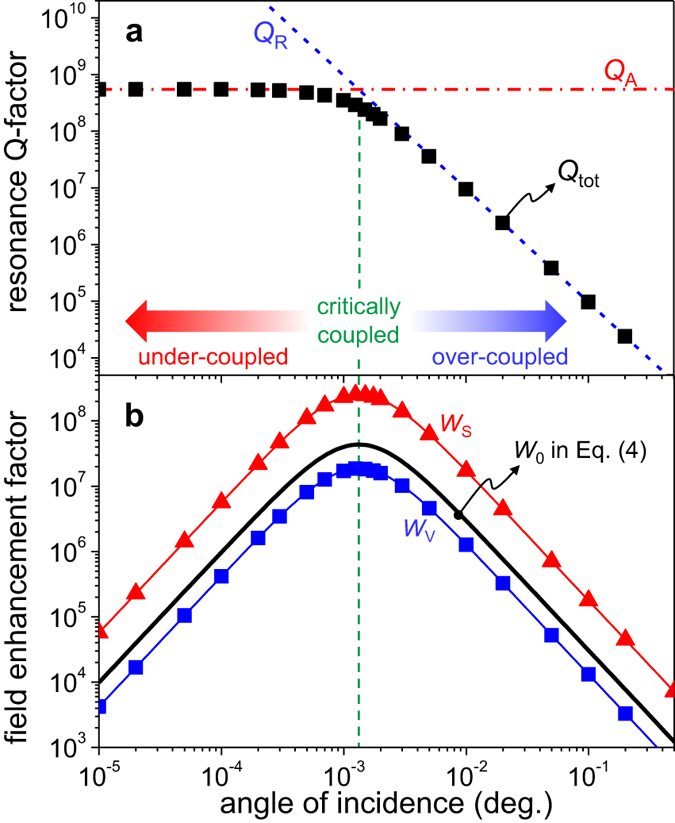
Field enhancement factors for critically coupled resonances in region A. (**a**) Partial and total resonance Q-factors. (**b**) Electric field intensity enhancement factors.

**Figure 5:**
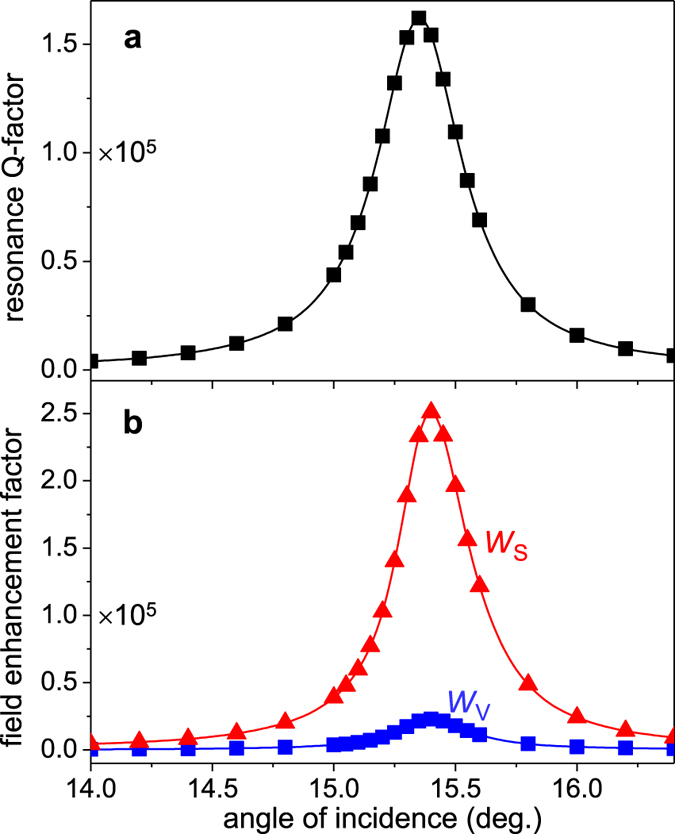
Field enhancement factors for over-coupled resonances in region B. (**a**) Total resonance Q-factor *Q*_tot_. (**b**) Electric field intensity enhancement factors. Note that *Q*_A_ = 4.2~4.4 × 10^8^ is much higher than *Q*_R_ ≈ *Q*_tot_ in region B, implying that the region B resonances are highly over-coupled.

We estimate resonance Q factors *Q*_A_, *Q*_R_, and *Q*_tot_ and field enhancement factors *W*_V_ and *W*_S_ for our simulated cases as shown in Figs 4 and 5 for the region A and B resonances, respectively. For resonance Q factor estimation, we use the absorbance analysis method established in^22^,^23^,^24^. The field enhancement factors by the definition in Eqs. (1) and (2) are directly obtained from the calculated field distributions due to the rigorous coupled-wave analysis.

Figure 4a shows the dependences of *Q*_A_, *Q*_R_, and *Q*_tot_ on the angle of incidence *θ* for the region A resonance. The radiation Q factor *Q*_R_ is increasing with decreasing angle of incidence because of the symmetry incompatibility as discussed previously. In contrast, the dissipation Q factor *Q*_A_ is almost constant because the modal field distribution does not remarkably change the portion of field energy inside the Si ridges with the change in *θ*. Therefore, the total Q factor *Q*_tot_ increases with *Q*_R_ for the large angle-of-incidence range of *θ* > 0.01° and becomes saturated at *Q*_A_ = 5.46 × 10^8^ for the small angle-of-incidence range of *θ* < 0.001°. Dependences of the field enhancement factors are quite distinguished from those of resonance Q factors as shown in Fig. 4b. Both *W*_V_ and *W*_S_ increase with increasing *Q*_tot_ for the large angle-of-incidence range of *θ* > 0.01°, they are maximized at *θ* = 0.00125° where *Q*_A_ = *Q*_R_, and they eventually show high slope decrease in the small angle-of-incidence range of *θ* < 0.001° although the total resonance Q factor is almost constant in this angle-of-incidence range.

We explain this non-trivial property of field enhancement factors on the basis of interference effects associated with generic optical Fano resonances. Using the coupled-mode theory of optical Fano resonances^23^,^25^, the energy stored in the resonance mode at the resonance center is expressed by


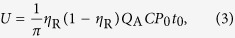


where *η*_R_ ≡ *γ*_R_/(*γ*_A_ + *γ*_R_) = *Q*_tot_/*Q*_R_ is the radiation probability of the resonance mode, *C* ≡ *γ*_1_/*γ*_R_ with *γ*_1_ denoting the partial radiation decay rate to the reflected wave is the relative strength of radiation coupling of the resonance mode with the reflected wave, *P*_0_ is the power delivered by the incident wave, and *t*_0_ ≡ 2*π*/*ω*_0_ is the optical cycle at the resonance center. According to the Lorentz reciprocity theorem for electromagnetic fields, the coupling strength constant *C* also can be interpreted as a relative coupling strength of the resonance mode with the incident wave. Field enhancement factors *W*_V_ and *W*_S_ are directly proportional to the ratio of the energy *U* stored in the resonance mode to the portion of the incoming energy *CP*_0_*t*_0_ coupled with the resonance mode for an optical cycle. Following this argument, we define a general field enhancement factor *W*_0_ such that


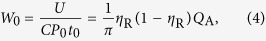


Equation (4) with the partial resonance Q factors *Q*_A_ and *Q*_R_ found in Fig. 4a quantitatively describes the dependence of *W*_V_ and *W*_S_ on the angle of incidence as shown in Fig. 4b.

It is obvious in Eq. (4) that the field enhancement does not simply increase with the total Q factor *Q*_tot_ but it has the maximum at the critical coupling condition where *η*_R_ = 1/2, or *Q*_R_ = *Q*_A_ = 2*Q*_tot_ equivalently. This is a result of a well-known effect of destructive interference of the resonant and non-resonant pathways in the reflected and transmitted waves. At the critical coupling condition, the destructive interference becomes strongest because the two contributions of the resonant and non-resonant pathways to the outgoing waves have the same intensities with *π* phase difference. This intensity and phase property is dictated by the time-reversal symmetry of the wave coupling processes^22^,^23^,^25^.

For under-coupled resonances with *η*_R_ < 1/2 and *Q*_R_ > *Q*_A_, intensity of the resonant contribution is not strong enough to cancel the non-resonant contribution and hence the light trapping effect is not as strong as for the critically coupled resonances. Alternatively, the field enhancement for an under-coupled resonance does not reach its obtainable maximum with a given *Q*_tot_ because the rate of radiative energy coupling from the incident wave to the resonance mode is slower than the internal damping rate due to the material dissipation. For highly under-coupled resonances with *η*_R_ ≪ 1/2 and *Q*_R_ ≫ *Q*_A_, Eq. (4) reduces to *W*_0_ ≈ (*Q*_A_/*Q*_R_)*Q*_tot_/*π*. Therefore, the field enhancement is suppressed by a factor *Q*_A_/*Q*_R_ (≪1) from that expected for lossless or highly over-coupled resonances. This explains the high-slope decrease of the field enhancement factors of the region A resonance without substantial decrease in *Q*_tot_ for the small angle-of-incidence range in Fig. 4b.

In the opposite cases of over-coupled resonances with *η*_R_ > 1/2 and *Q*_R_ < *Q*_A_, the resonant contribution dominates the outgoing wave intensities over that from the non-resonant contribution and hence the trapping effect becomes weaker than that for the critically coupled resonances. Alternatively, an over-coupled resonance can be considered to have an excessive radiation decay that exhausts the localized energy before it reaches its maximum limit for a given amount of material dissipation. Equation (4) for the general field enhancement factor reduces to *W*_0_ ≈ *Q*_tot_/*π* for highly over-coupled resonances with *η*_R_ ≫1/2 and *Q*_R_ ≪ *Q*_A_. Therefore, the field enhancement is simply proportional to the total Q factor. This explains the dependences of *W*_V_ and *W*_S_ on the angle of incidence for the region B resonance that is highly over coupled with *Q*_A_ ≫ *Q*_tot_ ≈ *Q*_R_. Figure 5a,b show the estimated *Q*_tot_ and field enhancement factors as functions of angle of incidence. We confirm that the volume and surface field enhancement factors (*W*_V_ and *W*_S_) are maximized at the angle of incidence corresponding to the *Q*_tot_ peak. Note that lossless cases is also considered as a highly over-coupled resonance because lossless resonances satisfy *Q*_A_ = ∞ » *Q*_R_.

Considering real experiments on ultrahigh-Q resonances and associated strong field enhancement, it is important to optimize the amount of the radiation damping of a resonance in accordance with the material dissipation of thin-film materials. High-index semiconductors, nitrides, and metal oxides such as Si, Ge, GaAs, Si3N_4_, TiO_2_, and HfO_2_ are common materials for resonant thin-film devices. The intrinsic dissipation of these materials is unavoidable and highly dependent on the film deposition and lithographic etching conditions that determine grain size, density of vacancies, and surface roughness. In addition to the material’s internal absorption, these film imperfections cause diffuse scattering that contributes to extinction coefficients in the order of 10^–5^ ~ 10^–3^ 26,27,28. Therefore, in order to obtain the strong field enhancement effect, device parameters such as fill factor and modulation depth of the pattern that primarily determine the radiation damping rate of a resonance mode should be properly optimized to satisfy the critical coupling condition for a given level of material dissipation. This factor is even more important in nanostructured plasmonic resonance systems including surface plasmonic biochemical sensors and nonlinear plasmonic metamaterials^29^,^30^. In plasmonic metals including Ag, Au, Al, and Cu, the ohmic absorption is high in the optical frequency domain and dense surface charges induce strong light scattering even from deep-subwavelength rough features on their surfaces.

## Conclusion

In conclusion, we address ultrahigh-Q resonances in a subwavelength Si grating. We show that a strongly modulated *c*-Si subwavelength grating supports embedded and quasi-embedded photonic bound states with resonance Q and field enhancement factors exceeding 10^8^. We find that in the presence of material dissipation the field enhancement associated with an embedded photonic bound state is not simply proportional to the resonance Q factor because of a particular interference effect generally involved in the resonant light trapping effect. Using the coupled-mode theory of general optical Fano resonances, we derive an analytic expression of the field enhancement factor in terms of the radiation probability and absorption Q factor of a bound state. We confirm quantitative agreement of this expression with rigorous numerical calculation results showing the field enhancement maximized at the critical condition where the radiation Q factor is identical to the absorption Q factor. Therefore, our results provide useful knowledge for designing practical resonance elements in various application areas including SERS-based molecular detection systems, cavity-QED problems, and nonlinear optical devices.

## Additional Information

**How to cite this article**: Yoon, J. W. *et al.* Critical field enhancement of asymptotic optical bound states in the continuum. *Sci. Rep.* **5**, 18301; doi: 10.1038/srep18301 (2015).
